# Synthesis, Crystal Structure, and Spectra Properties of the Cadmium (II) Complex with Bis(*N*-allylbenzimidazol-2-ylmethyl)benzylamine

**DOI:** 10.1155/2011/705989

**Published:** 2011-10-05

**Authors:** Huilu Wu, Jingkun Yuan, Ying Bai, Fan Kou, Fei Jia, Bin Liu

**Affiliations:** School of Chemical and Biological Engineering, Lanzhou Jiaotong University, Lanzhou 730070, China

## Abstract

A novel complex of cadmium (II) picrate (pic) with V-shaped ligand bis(*N*-allylbenzimidazol-2-ylmethyl)benzylamine (babb), with composition [Cd(babb)_2_](pic)_2_, was synthesized and characterized by elemental analyses and electrical conductivity, IR, and UV/visible spectra. The crystal structure of the complex has been determined by the single-crystal X-ray diffraction. In the complex, the coordination sphere around Cd (II) is distorted octahedral, six nitrogen atoms involved in coordination afforded by two tridentate ligand babb. Moreover, The DNA-binding properties of the ligand babb and Cd (II) complex were investigated by spectrophotometric methods and viscosity measurements, and the results suggest that they bind to DNA via an intercalation binding mode, and the Cd (II) complex shows higher affinity than the ligand.

## 1. Introduction

Benzimidazole is a typical heterocyclic ligand with nitrogen as the donor atom. It may mimic the histidine imidazole in coordination aspects and it is a component of biologically important molecules [1]. Due to the privileged structure [2], benzimidazoles and their derivatives have attracted great deal of interests because of their potential application in the area of drugs and pharmaceuticals such as antitumor [3], antiviral [4], anticancer [5], antimicrobial [6], antiprotozoal [7], and anti-inflammatory or analgesic activities [8]. Besides, this type of materials was distinctively studied for their reaction activities and roles as important intermediate in the inorganic and organic synthesis. Binding studies of small molecules to DNA are very important in the development of DNA molecular probes and new therapeutic reagents [9]. Transition metal complexes have attracted considerable attention as oxidation catalysts [10], probes in electron-transfer reactions involving metallo-proteins [11], and intercalators with DNA [12]. The studies about transition metal complexes contribute to further understanding irreplaceable role of transition metal in vivo [13].

In previous papers [14–17], we have investigated the coordinating ability of some kind of benzimidazoles ligands. In this paper, we have prepared and investigated the spectrum properties and crystal structure of the cadmium (II) complex with a novel V-shaped ligand bis(*N*-allylbenzimidazol-2-ylmethyl)benzylamine (babb). Besides, we also conduct the related research on the DNA-binding properties of this compound to investigate the mode of the complex bond to DNA.

## 2. Experimental

### 2.1. Materials and Instruments

The C, H, and N elemental analyses were determined using a Carlo Erba 1106 elemental analyzer. Electrolytic conductance measurements were made with a DDS-307-type conductivity bridge using 3 × 1^−3^ mol L^−1^ solutions in DMF at room temperature. The IR spectra were recorded in the 4000–400 cm^−1^ region with a Nicolet FT-VERTEX 70 spectrometer using KBr pellets. Electronic spectra were taken on a Lab-Tech UV Bluestar spectrophotometer. The fluorescence spectra were recorded on an LS-45 spectrofluorophotometer. 

 Calf thymus DNA (CT-DNA) and ethidium bromide (EB) were purchased from Sigma. All chemicals used were of analytical grade. All the experiments involving interaction of the ligand and the complexes with CT-DNA were carried out in doubly distilled water buffer containing 5 mM Tris and 50 mM NaCl and adjusted to pH 7.2 with hydrochloric acid. A solution of CT-DNA gave a ratio of UV absorbance at 260 and 280 nm of about 1.8–1.9, indicating that the CT-DNA was sufficiently free of protein [18]. The CT-DNA concentration per nucleotide was determined spectrophotometrically by employing an extinction coefficient of 6600 M^−1^ cm^−1^ at 260 nm [19].

 Absorption titration experiments were performed with fixed concentrations of the complex, while gradually increasing the concentration of CT-DNA. To obtain the absorption spectra, the required amount of CT-DNA was added to both compound solution and the reference solution to eliminate the absorbance of CT-DNA itself. From the absorption titration data, the binding constant (*K*_*b*_) was determined using the equation in [20]

(1) $$\frac{\left[\text{DNA}\right]}{\varepsilon_{a}-\varepsilon_{f}}=\frac{\left[\text{DNA}\right]}{\varepsilon_{b}-\varepsilon_{f}}+\frac{1}{K_{b}\left(\varepsilon_{b}-\varepsilon_{f}\right)},$$

where [DNA] is the concentration of CT-DNA in base pairs, *ε*_*a*_ corresponds to the extinction coefficient observed (*A*_obsd_/[M]), *ε*_*f*_ corresponds to the extinction coefficient of the free compound *ε*_*b*_, is the extinction coefficient of the compound when fully bound to CT-DNA, and *K*_*b*_ is the intrinsic binding constant. The ratio of slope to intercept in the plot of [DNA]/(*ε*_*a*_ − *ε*_*f*_) versus [DNA] gave the value of *K*_*b*_.

 EB emits intense fluorescence in the presence of CT-DNA, due to its strong intercalation between the adjacent CT-DNA base pairs. It was previously reported that the enhanced fluorescence can be quenched by the addition of a second molecule [21, 22]. The extent of fluorescence quenching of EB bound to CT-DNA can be used to determine the extent of binding between the second molecule and CT-DNA. The competitive binding experiments were carried out in the buffer by keeping [DNA]/[EB] = 1 and varying the concentrations of the compounds. The fluorescence spectra of EB were measured using an excitation wavelength of 520 nm, and the emission range was set between 550 and 750 nm. The spectra were analyzed according to the classical Stern-Volmer equation in [23]

(2) $$\frac{I_{0}}{I}=1+K_{\text{SV}}\left[Q\right],$$

where *I*_0_ and *I* are the fluorescence intensities at 599 nm in the absence and presence of the quencher, respectively, *K*_SV_ is the linear Stern-Volmer quenching constant, and [*Q*] is the concentration of the quencher. In these experiments, [CT-DNA] = 2.5 × 10^−3^ mol/L, [EB] = 2.2 × 10^−3^ mol/L.

 Viscosity experiments were conducted on an Ubbelodhe viscometer, immersed in a water bath maintained at 25.0 ± 0.1°C. Titrations were performed for the complexes (3–35 *μ*M), and each compound was introduced into CT-DNA solution (50 *μ*M) present in the viscometer. Data were presented as (*η*/*η*_0_)^1/3^ versus the ratio of the concentration of the compound to CT-DNA, where *η* is the viscosity of CT-DNA in the presence of the compound and *η*_0_ is the viscosity of CT-DNA alone. Viscosity values were calculated from the observed flow time of CT-DNA-containing solutions corrected from the flow time of buffer alone (*t*_0_), *η* = (*t* − *t*_0_) [24].

### 2.2. Compound Preparation

#### 2.2.1. Preparation of the Bis(N-allylbenzimidazol-2-ylmethyl)benzylamine (babb)

The precursor—bis(2-benzimidazol-2-ylmethyl)benzylamine (bbb) was synthesized following a slight modification of the procedure [25]. 7.34 g (20mmol) bbb with 1.56 g (40 mmol) potassium in 150 mL tetrahydrofuran followed by adding 4.84 g (40 mmol) allyl bromide. 

The resulting solution was concentrated and recrystallized from ethanol and the pale yellow block crystals were obtained [26]. Yield: 5.46 g (61%); m.p.: 113–115°C. Anal. calcd. for C_29_H_29_N_5_: C, 77.82; H, 6.53; N, 15.65. Found: C, 78.02; H, 6.35; N, 15.71%. IR data (KBr **ν*/*cm^−1^): 737 (*ν*_o−Ar_); 1265 (*ν*_C−N_); 1461 (*ν*_C=N_), 1614 (*ν*_C=C_).

#### 2.2.2. Preparation of [Cd(babb)_2_](pic)_2_

To a stirred solution of babb (223.5 mg, 0.5 mmol) in hot EtOH (10 mL) was added cadmium (II) picrate (142.15 mg, 0.25 mmol) in EtOH (5 mL). A yellow crystalline product which formed rapidly was filtered off, washed with EtOH and absolute Et_2_O, and dried in *vacuo*. The dried precipitate was dissolved in acetonitrile resulting in a yellow solution that was allowed to evaporate at room temperature. Pale yellow crystals suitable for X-ray diffraction studies were obtained after two weeks. Yield: 269 mg (67%). Anal. calcd. for C_70_H_62_CdN_16_O_14_ (MW 1463.76): C, 69.14; H, 5.80; N, 13.90. Found: C, 69.33; H, 5.71; N, 13.79%. Λ_M_ (DMF, 297 K): 135.67 S cm^2^ mol^−1^. IR data (KBr **ν*/*cm^−1^): 746 (*ν*_o−Ar_); 1288 (*ν*_C−N_); 1479 (*ν*_C=N_), 1633 (*ν*_C=C_).

#### 2.2.3. X-Ray Structure Determination

A suitable single crystal was mounted on a glass fiber and the intensity data were collected on a Bruker APEX-II CCD diffractometer with graphite-monochromated Mo-*K_*α*_* radiation (*λ* = 0.71073 Å) at 153 K. Date reduction and cell refinement were performed using SAINT programs [27]. The absorption corrections were carried out by the empirical method. The structure was solved by direct methods and refined by full-matrix least-squares against *F* ^2^ of data using SHELXTL software [28]. All H atoms were found in difference electron density maps and were subsequently refined in a riding-model approximation with C–H distances ranging from 0.93 to 0.97 Å and U_iso_  (H) = 1.2 U_eq_  (C). Basic crystal data, description of the diffraction experiment, and details of the structure refinement are given in Table 1. Selected bond distances and angles are presented in Table 2.

## 3. Results and Discussion

The cadmium complex is soluble in DMF, DMSO, and acetonitrile, but insoluble in methanolethanol and other organic solvents. The elemental analyses show that the composition is [Cd(babb)_2_](pic)_2_, which is the same with the theoretical ratio. A composition of molar conductance value shows the 1 : 2 electrolytes of the complex in DMF with those previously reported in the literature [29].

### 3.1. Crystal Structure

**Figure 1:**
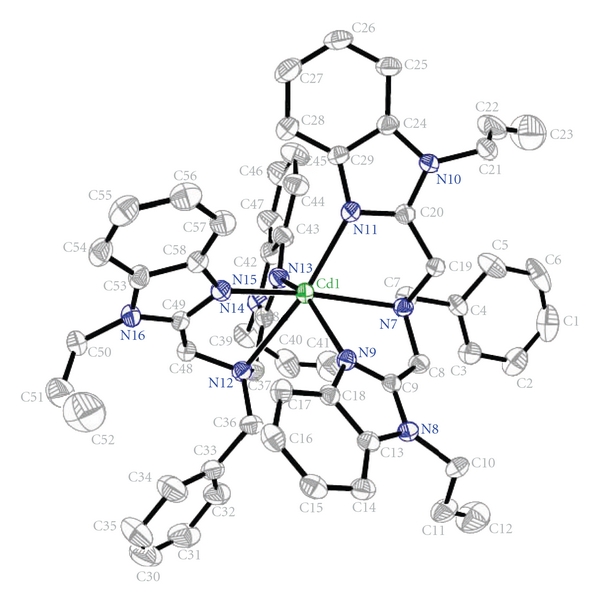
Molecular structure and atom-numberings of [Cd(babb)_2_]^2+^ with hydrogen atoms omitted for clarity.

The crystal structure of complex consists of discrete [Cd(babb)_2_]^2+^ cation and two picrate anions. The ORTEP structure of [Cd(babb)_2_]^2+^ with atom-numberings is shown in Figure 1.

The cadmium ion is six-coordinate with an N6 ligand set. The ligand babb acts as a tridentate N-donor. The coordination geometry of the Cd (II) may be best described as distorted octahedral with (N9, N11, N12, N13) from an equatorial plane. In the four selected nitrogen atoms, the maximum deviation distance (N13) from the least square plane calculated from the four N atoms is 0.321 (3) Å, which indicated four selected coordination nitrogen atoms almost in a plane. The bond angle of the two atoms (N7, N15) in axial positions is 166.37(10)° Å, and the distances deviation from the plane are 2.100 Å and 2.395 Å, respectively. Therefore, compared with a regular octahedron, it reflects a relatively distorted coordination octahedron around Cd (II) [30].

### 3.2. IR and UV-Visible Spectra

In the free ligand, a strong band is found around 1265 cm^−1^ along with a medium band at 1461 cm^−1^. By analogy with the assigned bands of imidazole, the former is attributed to *υ*_C−N_, while the other one is *υ*_C=N_ [31–33]. The location of the two bands occurred slightly migration in the complex, which implies direct coordination of all four imine nitrogen atoms to Cd (II) [34]. Information regarding the possible bonding modes of the picrate and benzimidazole rings also can be obtained from the IR spectra [35]. 

DMF solutions of the ligand babb and the complex show, as expected, that the UV bands of babb (279 nm) are only marginally blue-shifted in the complex, which shows clear evidence of C=N coordination to cadmium. The absorption bands are assigned to *π*→π* (imidazole) [36]. This conclusion is confirmed by the result of the crystal structure analysis.

### 3.3. Absorption Titration

The absorption titration experiment was carried out to investigate the binding affinity of the complexes with CT-DNA. A complex bound to DNA through intercalation is characterized by hypochromism in absorbance and red shift in wavelength, due to the intercalation mode involving a strong stacking interaction between the aromatic chromophore and the DNA base pairs [37]. A certain amount of hypochromism is commonly consistent with the strength of the intercalative interaction [38–40]. The intrinsic binding constants *K*_*b*_ of the complexes with DNA were obtained by monitoring the changes in absorbance at 270–280 nm for the complex with increasing concentration of DNA. The absorption spectral titration of the complex binding to DNA was performed by increasing a certain amount of DNA (5 *μ*L) to complex in Tris-HCl buffer. The sample solution was scanned in the range of 200–500 nm. The constant (*K*_*b*_) was obtained by the following equation [20]: [DNA]/(*ε*_*a*_ − *ε*_*f*_) = [DNA]/(*ε*_*b*_ − *ε*_*f*_) + 1/*K*_*b*_(*ε*_*b*_ − *ε*_*f*_), apparent absorption coefficient *ε*_*a*_, *ε*_*f*_, and *ε*_*b*_ correspond to *A*_obsd_/[M], the extinction coefficient of the free complex and the extinction coefficient of the complex when fully bound to DNA, respectively. In plots of [DNA]/(*ε*_*a*_ − *ε*_*f*_) versus [DNA], *K*_*b*_ is given by the ratio of slope to the intercept.

**Figure 2:**
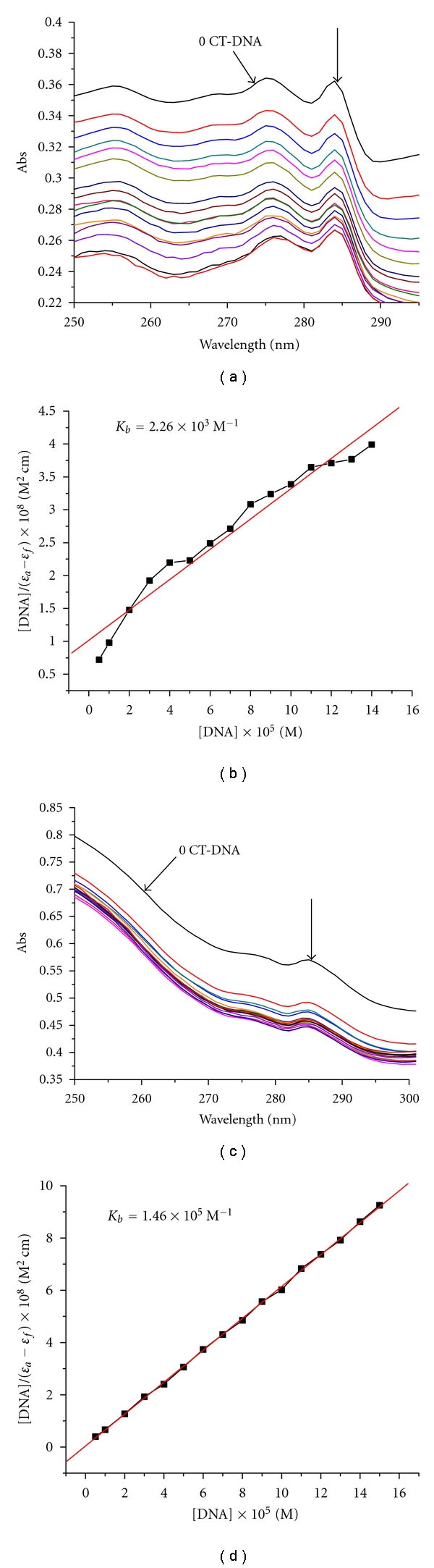
Absorption spectra of the ligand babb (a), Cd (II) complex (c) (3 × 10^−5^ mol L^−1^) in the absence and presence of increasing amounts of DNA (0–15 × 10^−5^ mol L^−1^) in 5 mmol L^−1^ Tris-HCl/50 mmol L^−1^ NaCl buffer (pH = 7.2). The arrow shows absorbance changes on increasing DNA concentration. Plot of [DNA]/(*ε*_*a*_ − *ε*_*f*_) versus [DNA] for absorption titration of DNA with the ligand babb (b), Cd (II) complex (d).

The absorption spectra of the ligand babb and Cd (II) complex in the absence and presence of DNA at different concentrations are given in Figure 2. With increasing DNA concentrations, the hypochromisms are 18.26% at 275 nm for babb and 50.29% at 280 nm for Cd (II) complex. The *λ*_max _  for ligand babb and Cd (II) complex increased from 275 to 276 nm, 276 to 278 nm, respectively. The *K*_*b*_ values of babb, Cd (II) complex were 2.26 × 10^3^ M^−1^ (*R* = 0.98 for 15 points), 1.46 × 10^5^ M^−1^ (*R* = 0.99 for 16 points), respectively. The results may be suggested that the ligand babb and Cd (II) complex bind to DNA via the intercalation mode, involving strong *π*→π* stacking interactions between benzimidazole rings of the compounds and DNA base pairs, and the binding strength of Cd (II) complex is greater than the ligand.

### 3.4. EB Competitive Experiment

No luminescence was observed for the complex at room temperature in any organic solvent or in the presence of CT-DNA. So the binding of complexes with CT-DNA cannot be directly presented in the emission spectra. Therefore, competitive EB binding studies could be undertaken in order to examine the binding of each complex with DNA. EB (ethidium bromide) is a conjugate planar molecule. Its fluorescence intensity is very weak in solvent, but it is greatly increased when EB is specifically intercalated into the base pairs of double-stranded DNA. In previous studies, the fluorescent light could be quenched by the addition of the complex which can compete with EB to bind with DNA. This is a proof that the complexes intercalate to base pairs of DNA [41]. The Stern-Volmer quenching constant *K*_SV_ is often used to evaluate the quenching efficiency for each complex and determined by the classical Stern-Volmer equation [23]:

(3) $$\frac{I_{0}}{I}=1+K_{\text{SV}}\left[Q\right].$$

*I*_0_ and *I* are the fluorescence intensities in the absence and presence of the quencher, respectively, and [*Q*] is the concentration of the complex. 

**Figure 3:**
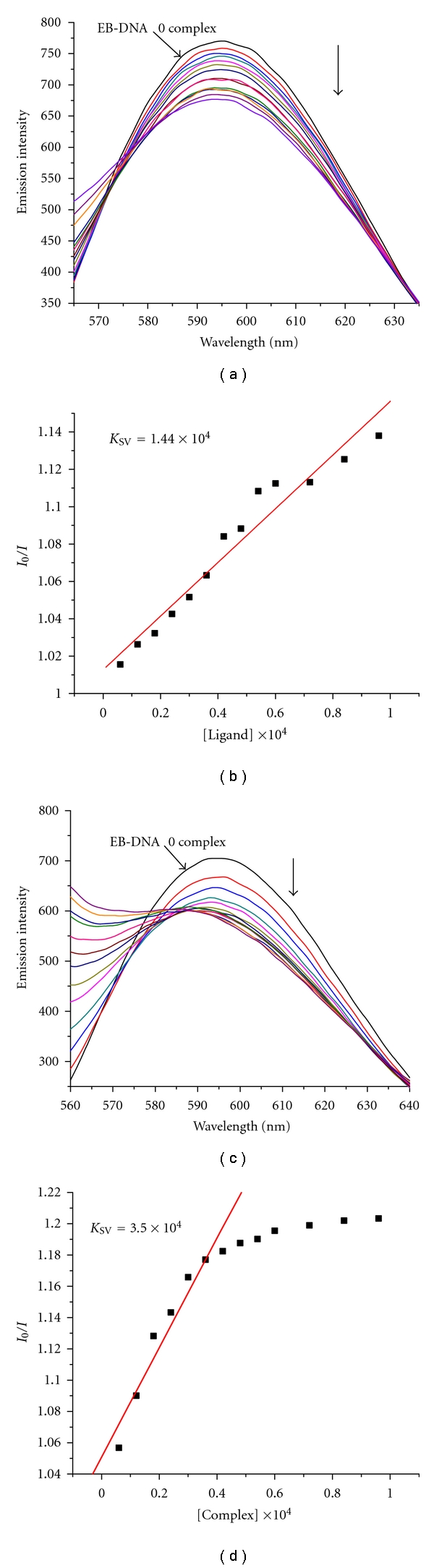
Emission spectra of EB bound to CT-DNA in the presence of the ligand babb (a), Cd (II) complex (c), [Complex] = 3 × 10^−5^ M; *λ*_ex_ = 520 nm. The arrows show the intensity changes upon increasing concentrations of the complex. Fluorescence quenching curves of EB bound to CT-DNA by the ligand babb (b), Cd (II) complex (d). (Plots of *I*_0_/*I* versus [Complex].)

It was clear that the fluorescence intensity of EB-DNA system greatly decreases upon the addition of the complexes (5 *μ*L) gradually. The fluorescence quenching of EB-DNA by the complex is shown in Figure 3. The Stern-Volmer constant *K*_SV_ is obtained as the slope of *I*_0_/*I* versus complex linear plot. From Figure 3, the *K*_SV_ values for the ligand babb and Cd (II) complex are 1.44 × 10^4^ M^−1^ (*R* = 0.98 for 13 points) and 3.50 × 10^4^ M^−1^ (*R* = 0.98 for 7 points in the linear part), respectively. The results suggest that the ligand babb and Cd (II) complex can compete for DNA-binding sites with EB and displace EB from the EB-DNA system [42], which is usually characteristic of the intercalative interaction of compounds with DNA [43]. Additionally, The data suggest that the interaction of the Cd (II) complex with CT-DNA is stronger than that of the ligand babb, which is consistent with the above absorption spectral results. 

### 3.5. Viscosity Measurement

**Figure 4:**
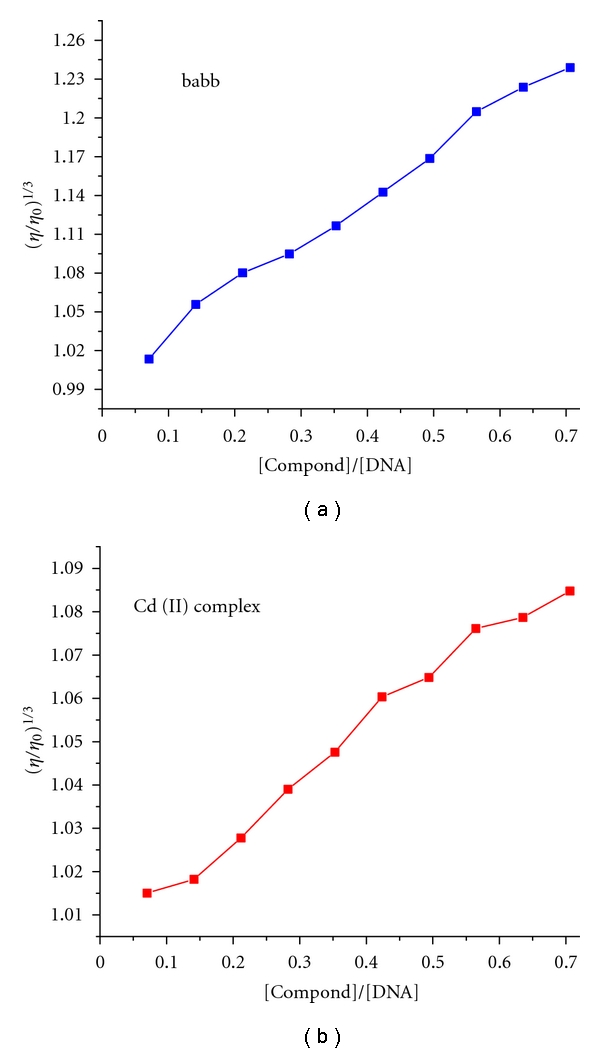
Effect of increasing amounts of the ligand babb (a), Cd (II) complex (b) on the relative viscosity of CT-DNA at 25 (±0.1)°C in 5 mmol L^−1^ Tris-HCl buffer (pH = 7.2, [DNA] = 5 × 10^−5^ M).

Further clarification of the interactions between the Cd (II) complex and DNA was carried out by viscosity measurements. Due to its sensitivity to the change of length of DNA, viscosity measurement may be the most effective means to study the binding mode of complex to DNA [22]. A significant increase in the viscosity of DNA on the addition of the compounds indicates the intercalative mode of binding to DNA. In contrast, complex that binds in the DNA grooves by partial and/or nonclassical intercalation causes less pronounced (positive or negative) or no change in DNA solution viscosity [40]. Titrations were performed for the compounds (3–35 **μ**M), and the complex was introduced into the CT-DNA solution (50 **μ**M) present in the viscometer. Viscosity values were calculated from the observed flow time of CT-DNA containing solutions corrected from the flow time of buffer alone (*t*_0_), *η* = (*t* − *t*_0_)/*t*_0_ [44]. Data were presented as (*η*/*η*_0_)^1/3^ versus the ratio of the concentration of the complex to CT-DNA, where *η* is the viscosity of CT-DNA in the presence of the complex and *η*_0_ is the viscosity of CT-DNA alone. Viscosity measurements were carried out on CT-DNA by varying the concentration of the compound.  Figure 4 shows the ligand babb and Cd (II) complex cause increase in the relative viscosity of DNA. This may be explained by the fact that they both can intercalate the adjacent DNA base pairs, leading to an increase in the separation of base pairs at intercalation sites and, thus, an increase in overall DNA contour length. So the results demonstrate that the complex could bind to DNA by intercalation mode, which is consistent with the above absorption and fluorescence spectral results.

## 4. Conclusions

In this paper, a new ligand babb and its Cd (II) complex have been synthesized and characterized. The DNA-binding properties of the ligand babb and Cd (II) complex were investigated by electronic absorption, fluorescence, and viscosity measurements. The results indicate that they both can bond to CT-DNA in an intercalation mode and the Cd (II) complex shows higher affinity than the free ligand, which can be attributed to the more planar structure owing to upon coordination to the metal. The results can provide the evidence for designing the drugs and pharmaceuticals on a molecular level and warrant further in vivo experiments and pharmacological assays.

##  Supporting Information 

Crystallographic data (excluding structure factors) for the structure in this paper have been deposited with the Cambridge Crystallographic Data Centre as supplementary publication CCDC 826633. Copies of the data can be obtained, free of charge, on application to the CCDC, 12 Union Road, Cambridge CB2 1EZ, UK. Tel: +44-01223-762910; fax: +44-01223-336033; e-mail: deposit@ccdc.cam.ac.uk or http://www.ccdc.cam.ac.uk/.

## Figures and Tables

**Table 1:** Crystallographic data and data collection parameters for [Cd(babb)_2_](pic)_2_.

Complex	[Cd(babb)_2_](pic)_2_
Molecular formula	C_70_H_62_CdN_16_O_14_
Molecular weight	1463.76
Crystal system	Triclinic
Space group	P-1
a (Å)	13.903 (4)
b (Å)	14.146 (4)
c (Å)	19.330 (5)
*α* (°)	90.595 (3)
*β* (°)	110.890 (3)
*γ* (°)	106.325 (3)
*V *(Å^3^)	3382.5 (16)
Z	2
*ρ*_cald_ (mg m^−3^)	1.437
*F*(000)	1508
Crystal size (mm)	0.40 × 0.38 × 0.30
**θ** range for data collection (°)	2.25 to 25.00
*h*/*k*/*l* (max, min)	−16, 16/−15, 16/ −22, 22
Reflections collected	20103
Independent reflections	11730 [*R*(int) = 0.0253]
Completeness to theta = 25.00	98.4%
Refinement method	Full-matrix least- squares on *F* ^2^
Data/restraints/parameters	11730/49/910
Goodness-of-fit on *F* ^2^	1.008
Final *R*1, *wR*2 indices [*I* > 2*σ*(*I*)]	0.0473, 0.1117
*R*1, *wR*2 indices (all data)	0.0682, 0.1249
Largest differences peak and hole (e Å^−3^)	0.986 and −0.537

**Table 2:** Selected atomic distances (Å) and bond angles (°) for the complex.

Bond distances			
Cd(1)–N(15)	2.248 (3)	Cd(1)–N(13)	2.319 (3)
Cd(1)–N(11)	2.281 (3)	Cd(1)–N(7)	2.556 (3)
Cd(1)–N(9)	2.307 (3)	Cd(1)–N(12)	2.673 (3)

Bond angles			

N(15)–Cd(1)–N(11)	109.41 (11)	N(9)–Cd(1)–N(7)	70.12 (10)
N(15)–Cd(1)–N(9)	96.74 (11)	N(13)–Cd(1)–N(7)	92.25 (11)
N(11)–Cd(1)–N(9)	106.65 (11)	N(15)–Cd(1)–N(12)	70.80 (11)
N(15)–Cd(1)–N(13)	100.72 (12)	N(11)–Cd(1)–N(12)	165.10 (10)
N(11)–Cd(1)–N(13)	98.24 (11)	N(9)–Cd(1)–N(12)	87.96 (10)
N(9)–Cd(1)–N(13)	142.69 (11)	N(13)–Cd(1)–N(12)	67.50 (10)
N(15)–Cd(1)–N(7)	166.37 (10)	N(7)–Cd(1)–N(12)	111.11 (9)
N(11)–Cd(1)–N(7)	72.40 (10)		
